# Clinical Utility of Expanded Carrier Screening: Reproductive Behaviors of At-Risk Couples

**DOI:** 10.1007/s10897-017-0160-1

**Published:** 2017-09-27

**Authors:** Caroline E. Ghiossi, James D. Goldberg, Imran S. Haque, Gabriel A. Lazarin, Kenny K. Wong

**Affiliations:** 1 California State University Stanislaus, 1 University Cir, Turlock, CA 95382 USA; 2 Counsyl, 180 Kimball Way, South San Francisco, 94080 CA USA; 3 Present Address: Palo Alto Medical Foundation, Palo Alto, CA USA; 4 Present Address: Freenome, South San Francisco, CA USA

**Keywords:** Expanded carrier screening, Preimplantation genetic diagnosis, Prenatal diagnosis, Clinical utility

## Abstract

Expanded carrier screening (ECS) analyzes dozens or hundreds of recessive genes to determine reproductive risk. Data on the clinical utility of screening conditions beyond professional guidelines are scarce. Individuals underwent ECS for up to 110 genes. Five-hundred thirty-seven at-risk couples (ARC), those in which both partners carry the same recessive disease, were invited to participate in a retrospective IRB-approved survey of their reproductive decision making after receiving ECS results. Sixty-four eligible ARC completed the survey. Of 45 respondents screened preconceptionally, 62% (*n* = 28) planned IVF with PGD or prenatal diagnosis (PNDx) in future pregnancies. Twenty-nine percent (*n* = 13) were not planning to alter reproductive decisions. The remaining 9% (*n* = 4) of responses were unclear. Of 19 pregnant respondents, 42% (*n* = 8) elected PNDx, 11% (*n* = 2) planned amniocentesis but miscarried, and 47% (*n* = 9) considered the condition insufficiently severe to warrant invasive testing. Of the 8 pregnancies that underwent PNDx, 5 were unaffected and 3 were affected. Two of 3 affected pregnancies were terminated. Disease severity was found to have significant association (*p* = 0.000145) with changes in decision making, whereas guideline status of diseases, controlled for severity, was not (*p* = 0.284). Most ARC altered reproductive planning, demonstrating the clinical utility of ECS. Severity of conditions factored into decision making.

**Electronic supplementary material:**

The online version of this article (10.1007/s10897-017-0160-1) contains supplementary material, which is available to authorized users.

## Introduction

Carrier screening identifies couples at increased risk of having a child with a genetic disease and enables them to consider alternative reproductive options. Those who do not make alternative reproductive decisions based on their carrier status may still use this knowledge to prepare for the birth of an affected child and potentially facilitate early intervention (Edwards et al. 2015; Nazareth et al. 2015).

Historically, carrier screening programs targeted a small number of diseases that are highly prevalent in an ethnic-defined population. More recently developed, expanded carrier screening (ECS) assesses risk for dozens or hundreds of diseases across all populations, also known as pan-ethnic, or universal screening (Umbarger et al. 2013). The American College of Obstetricians and Gynecologists (ACOG) recently recognized ECS as an acceptable screening strategy (ACOG Committee Opinion No. 690 2017).

Despite widespread adoption of ECS by many providers, statements from professional organizations suggest additional research is needed (Henneman et al. 2016; Umbarger et al. 2013) in particular, citing the lack of data regarding reproductive outcomes of couples that undergo ECS. Studies of outcomes after population-based carrier screening initiatives for a limited number of disorders have consistently found a reduced incidence of the disease of interest due to the decisions made by the at-risk couples (ARC). Tay-Sachs disease incidence fell by 90% in the Ashkenazi Jewish population due to high screening uptake over the course of multiple decades (Kaback 2000). Screening for thalassemia in Mediterranean and Chinese populations has resulted in similar declines (Cao et al. 2002; Kaback 2000; Liao et al. 2005).

Decision-making among couples at risk for children affected by cystic fibrosis (CF) has been assessed in several studies. Many of the studies only included pregnant couples and often parents who had an affected child. While these studies indicated that the majority of couples would pursue prenatal diagnosis for the condition in question (Ioannou et al. 2015; Sawyer et al. 2006; Scotet et al. 2008), having a previously affected child or relative may influence reproductive decision making. Therefore, while decisions of carrier couples in this context are well-characterized, they do not represent the majority experience of ARC who are unlikely to have personal experience with CF prior to learning their carrier status. (Ioannou et al. 2015).

The available studies that are focused on CF screening in the general population, not based on family history or known carrier status, do demonstrate effectiveness in reducing the incidence of the condition. In the United States, a study in Massachusetts demonstrated a decrease in CF incidence following the screening recommendations by ACOG, American College of Medical Genetics and Genomics (ACMG) and the National Institutes of Health (Hale et al. 2008). Several studies have been conducted outside of the US. Of significance is a five-year study in Edinburgh, UK that showed a drop in CF incidence following the implementation of the screening recommendation (Cunningham and Marshall 1998). A three-year study of a screening program in Australia identified nine ARC, six that were pregnant at the time of screening. All six couples elected prenatal diagnosis, and three preconception couples elected to pursue in vitro fertilization (IVF) with preimplantation genetic diagnosis (PGD) (Massie et al. 2009).

Literature regarding the clinical utility of ECS panels is only beginning to emerge. One recent study by Franasiak et al. (2016) focused on the clinical decision-making of infertile couples found to be carriers through ECS testing as part of their infertility work-up. Eight couples were identified as at-risk carriers and elected to pursue PGD as part of their IVF treatment, indicating that the results of the carrier screening affected clinical decision making in all cases, though the authors noted that not all of the couples completed the planned treatment.

The purpose of this study was to learn about the reproductive decisions of ARC as identified by ECS from a nationwide population. This paper will describe the experience of these ARC after they received their ECS results, characterize their reproductive decisions, and identify factors associated with their decision making.

## Materials and Methods

This was a mixed-methods retrospective study in which participants were invited to self-report their experience and outcomes. The study was approved by the California State University Stanislaus Institutional Review Board, Protocol #1516–007.

### Participants

Participants were selected from those receiving expanded carrier screening (Foresight™ Carrier Screen) through Counsyl (South San Francisco, CA), a molecular diagnostics laboratory. The test detected carrier status in up to 110 genes, either by targeting 417 predefined disease-causing mutations or by next-generation exonic sequencing and pathogenicity interpretation for novel sequence variants. Test orders required physician authorization, typically a specialist in obstetrics, reproductive endocrinology, maternal-fetal medicine, or clinical genetics. Carrier screening was voluntary, and consent to research was included within the general consent form, with the option to request exclusion. Post-test genetic counseling was made available at no additional cost to all individuals tested.

Conditions included in the ECS panel range in severity. A method for severity classification divided diseases into four groups, from most to least impactful: profound, severe, moderate, and mild (Lazarin et al. 2014). In this classification algorithm, disease characteristics are organized into tiers based on their impact to the affected individual. Severity is assigned to a disease based a combination of the number of characteristics present in disease, and the tier ranking of those characteristics (Lazarin et al. 2014).

At-risk couples (ARC) were defined as self-identified reproductive partners in which both were identified as carriers for the same profound, severe, or moderate autosomal recessive condition. ARC identified by the laboratory between April 2014 and August 2015 were selected for inclusion in the study if contact information was available, neither individual had requested exclusion from research, and neither known personal carrier status of genetic disease nor testing for gamete donor candidacy was reported on the test requisition.

Couples at risk for a mild condition (e.g. pseudocholinesterase deficiency) or couples in which the female carried an X-linked condition (e.g. fragile X syndrome) were excluded. The former was decided in order to focus the study on diseases with greatest clinical impact, while the latter was meant to ensure that the risk for an affected child was consistent across couples, for autosomal recessive diseases, 25%.

### Instrumentation

An investigator-derived survey comprised of 33 questions was deployed online through SurveyGizmo (Boulder, CO), a survey research tool providing HIPAA-compliant privacy and security features. The survey included logic to skip or display certain questions based on each individual’s answer about pregnancy status and reproductive decisions. The survey questions were designed to elicit ARC’s experience with ECS, their reproductive choices, and future plans after receiving their ECS results. Choices assessed included in vitro fertilization (IVF) with preimplantation genetic diagnosis (PGD), prenatal diagnosis such as chorionic villus sampling (CVS) or amniocentesis, termination of pregnancy, adoption, gamete donation, no longer planning to have children, or (as free text) other changes in reproductive plans. Additionally, respondents could indicate if they were not planning to pursue any alternative options. Questions also included the condition for which they were found to be carriers, reasons for pursuing carrier screening, utilization of and satisfaction with genetic counseling, pregnancy history, and demographics. The survey consisted of multiple choice questions to capture categorical variables as quantitative data, as well as open-ended questions about participants’ experiences. Responses to the survey were anonymous. The full survey is provided in the supplemental information.

### Procedures

Data were collected from two sets of ARC. The first set (*n* = 465) were first invited by email, followed by a reminder email and/or a SMS text. A second set (*n* = 72) without email or mobile phone contact information on file were invited via paper mail. Upon survey completion, participants were given the option to enter their email address in a drawing for Amazon.com gift cards.

### Data Analysis

Survey data were analyzed in SPSS (IBM Corporation, Armonk, NY). Descriptive statistics were generated to characterize the trends in the data. Data categories were collapsed to 2 × 2 contingency tables and analyzed using Fisher’s exact test. To control for multiple hypothesis testing, the significant *p*-value was adjusted using the Bonferroni correction.

Free text responses were analyzed in Excel matrices (Microsoft Corporation, Redmond, WA). An open coding framework was used to identify broad themes in the participants’ experiences, decision-making rationale, and future plans. The coding was performed by a single investigator. The free text responses and corresponding coding decisions were circulated to the authors for review. While the qualitative analysis was not the focus of this study, these data were used to enrich the quantitative analysis and provide patient perspective and commentary on the trends seen in the statistics.

## Results

**Fig. 1:**
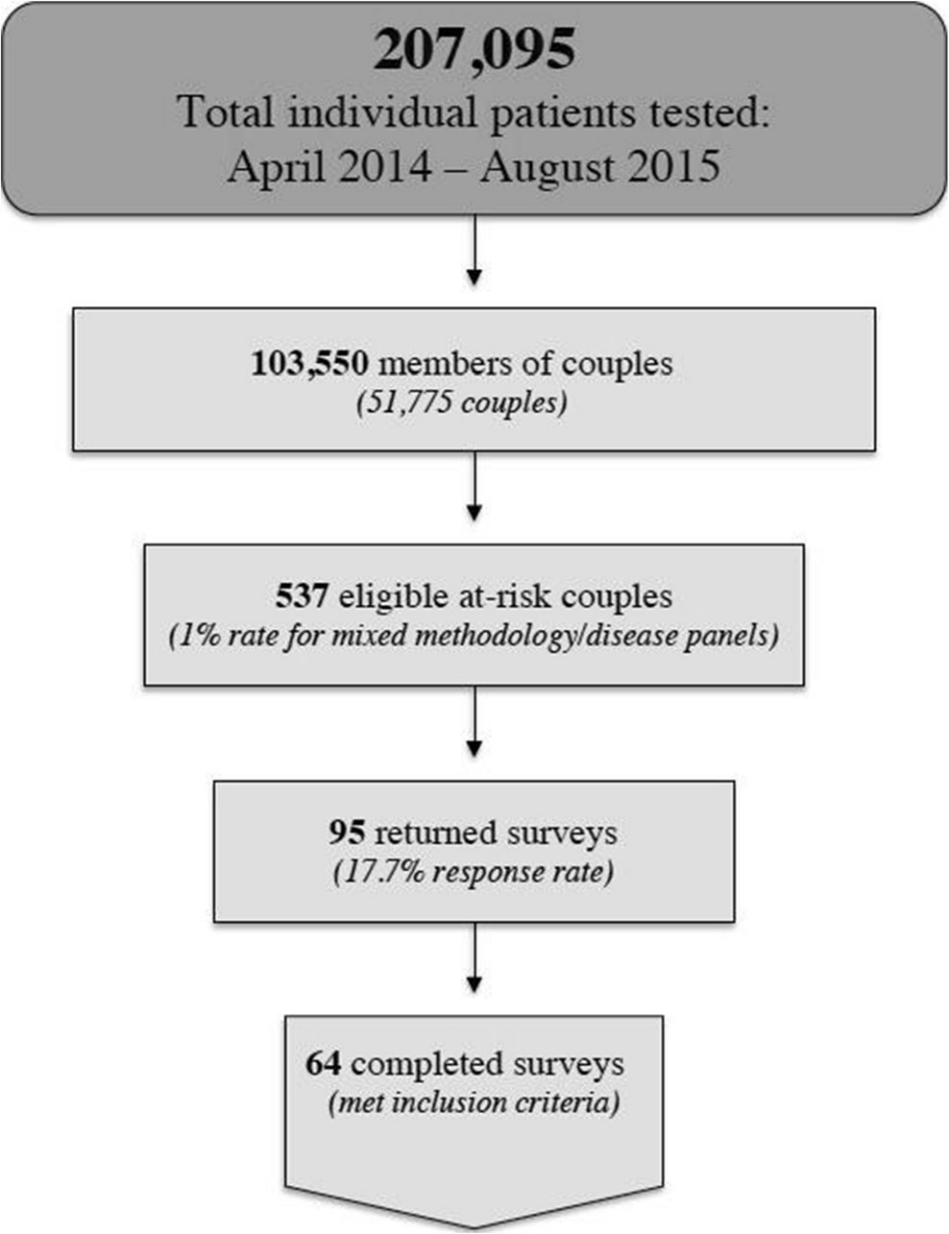
Participant inclusion process. *Illustration of total individual patients tested to yield final number of responses meeting inclusion criteria*

Of 207,095 individual patients tested for ECS between April 2014 and August 2015 (either genotyping or sequencing-based, and with a mix of selected diseases), 103,550 notified Counsyl that they were part of a couple and received merged reports. Of these 51,775 couples, our eligibility criteria yielded 537 ARC for possible participation; 465 could be contacted by SMS or email, and the remaining 72 could only be contacted through postal mail (Fig. 1).

The online survey received an 18% (*n* = 86) total response rate including 16 partial responses in which the respondent either declined consent and was exited from the survey, or provided consent but exited the survey prior to reaching the end. Fifteen percent (*n* = 70) completed the survey. Of the 72 paper surveys mailed via post, 13% (*n* = 9) were returned completed. Completion rate was not significantly different between methods (*p* = 0.72).

Of 79 completed surveys, three were excluded because the respondents did not report the condition for which they were both carriers, instead choosing either “I don’t recall” or “My partner and I were not carriers for the same condition.” An additional 12 that reported a family history of the condition for which they were found to be carriers were excluded from this analysis.

The demographic data of the remaining 64 participants are summarized in Tables 1 and 2. Respondents and their partners were predominantly Caucasian (76% and 70% respectively, selecting this option), educated (89% and 77% respectively, with bachelor’s degree or higher), with annual household incomes exceeding $100,000 (68%). The majority of female partners were 25–34 years of age (81%), had no children at the time of responding to the survey (62%), and had no history of miscarriage (69%). Most ARC had carrier screening as part of a fertility work-up (53%) with other reasons for carrier screening including routine screening (31%), ethnicity-based screening (6.3%), prior miscarriages or stillbirth (4.7%), ultrasound anomalies (3.1%), and consanguinity (1.6%). ARC reported having ECS on the recommendation of a healthcare provider (86%), and almost all pursued genetic counseling after receiving results (95%), the majority through Counsyl’s services (61%).

**Table 1:** Participant demographic information (*N* = 64)

Characteristic	*n*	Categories	Responses, n	Percentage
Female partner’s age	64	18–24	2	3.1%
		25–34	52	81%
		35–44	10	16%
Number of children	63	None	39	62%
		1	21	33%
		2+	3	4.8%
Past miscarriages	64	Yes	20	31%
		None	44	69%
Ethnicity	59 *(multiple selections allowed)*	European/Mixed Caucasian	45	66%
		Ashkenazi Jewish	11	16%
		Asian	4	5.9%
		African American	3	4.4%
		Others	5	7.4%
Partner’s ethnicity	58 *(multiple selections allowed)*	European/Mixed Caucasian	41	64%
		Ashkenazi Jewish	12	19%
		Asian	4	6.3%
		African American	3	4.7%
		Others	4	6.3%
Religious affiliation	64	None	17	27%
		Catholic	14	22%
		Protestant	13	20%
		Jewish	11	17%
		Others	5	7.8%
		Decline to state	4	6.3%
Partner’s religious affiliation	63	None	23	37%
		Protestant	12	19%
		Jewish	12	19%
		Catholic	9	14%
		Others	3	4.8%
		Decline to state	4	6.3%
Highest level of education	64	High school/ vocational school	3	4.7%
		Some college/ associate degree	4	6.3%
		Bachelor degree	23	36%
		Graduate degree	34	53%
Partner’s highest level of education	64	High school/ vocational school	5	7.8%
		Some college/ associate degree	10	16%
		Bachelor degree	19	30%
		Graduate degree	30	47%
Annual household income	63	<$49,999	7	11%
		$50,000 - $74,999	5	7.9%
		$75,000 - $99,999	8	13%
		$100,000 -$150,000	23	37%
		>$150,000	20	32%

**Table 2:** Motivations and circumstances for pursuing carrier screening (*N* = 64)

Characteristic	*n*	Categories	Responses	Percentage
Reason for screening	64	Fertility work-up	34	53%
		Routine screening	20	31%
		Multiple miscarriages/stillbirth	3	4.7%
		Ethnicity	4	6.3%
		Consanguinity	1	1.6%
		Ultrasound anomaly	2	3.1%
Initiator of screening	63	Healthcare provider	54	86%
		Patient requested	9	14%
Length of time since receiving results	64	1–3 months	11	17%
		3–6 months	13	20%
		6–9 months	8	13%
		>9 months	32	50%
Genetic counseling (GC) services	64	Counsyl GC	37	58%
		Local GC	22	34%
		Other provider	2	3.1%
		None	3	4.7%
Disease severity classification	64	Moderate	19	30%
		Severe	34	53%
		Profound	11	17%
Pregnancy status at time of receiving results	64	Pregnant	19	30%
		Preconception	45	70%

Sixty participants provided an interpretable answer about their actions or planned actions following results receipt. The remaining four participants either declined to answer the question or did not supply an interpretable response (e.g. selecting “other” without additional explanation when asked about their plans or selecting/indicating contradictory options.) The reproductive decisions reported by ARC were collapsed into two categories: action (36/60) or no action (24/60) based on the ECS results, with the unclear responses (4/64) excluded from the statistical analysis. Actions reported by this group included IVF with PGD (*n* = 22) and prenatal diagnosis (*n* = 14).

Potential associations between alternative reproductive options and disease severity, pregnancy status (non-pregnant ARC have more available options), and demographic factors were assessed (Table 3). After Bonferroni correction, the required threshold for significance was .05/10 = *α* = .005.

**Table 3:** Associations with reproductive decisions

Disease severity categorization: moderate vs. severe/profound			
	Moderate	Severe/profound	Total
Action taken	4	32	36
No action	14	10	24
Total	18	42	*p* = 0.000145*
Disease severity categorization: severe vs. profound			
	Severe	Profound	Total
Action taken	24	8	32
No action	8	2	10
Total	32	10	*p* = 1.00
Diseases with universal screening guidelines (cystic fibrosis, spinal muscular atrophy) vs. other severe/profound diseases			
	Universal screening	No screening guidelines	Total
Action taken	14	18	32
No action	2	8	10
Total	16	26	*p* = 0.270
Diseases with universal or ethnicity - based screening guidelines (e.g., cystic fibrosis, Gaucher disease, beta-thalassemia) vs. other severe/profound diseases			
	Screening Guidelines	No screening guidelines	Total
Action taken	17	15	32
No action	3	7	10
Total	20	22	*p* = 0.284
Biotinidase deficiency vs. other severe/profound diseases			
	Biotinidase deficiency	Other severe/profound diseases	Total
Action taken	2	30	32
No action	5	5	10
Total	7	35	*p* = 0.0049*
Pregnancy status at time of receiving ECS results: preconception vs. prenatal			
	Preconception	Prenatal	Total
Action taken	28	8	36
No action	13	11	24
Total	41	19	*p* = 0.088
Prior children (*N* = 59)			
	Prior children	No children	Total
Action taken	11	24	35
No action	12	12	24
Total	23	36	*p* = 0.181
History of miscarriage			
	History of miscarriage(s)	No prior miscarriage(s)	Total
Action taken	11	25	36
No action	8	16	24
Total	19	41	*p* = 1.00
Level of education: ARC with one or more graduate degree vs. ARC with bachelor degrees or below			
	Graduate degree	Bachelor degree or below	Total
Action taken	25	11	36
No action	15	9	24
Total	40	20	p = 0.590
Annual household income: $100,000 or more vs. less than $100,000 (*N* = 59)			
	$100,000+	<$100,000	Total
Action taken	22	14	36
No action	19	4	23
Total	41	18	*p* = 0.093

*Summary of statistical analysis: Data on these variables were collapsed into 2 × 2 contingency tables and analyzed using Fisher’s exact. Demographics and fertility history were collapsed to the majority category* versus *all other categories as described below. Action taken based on ECS results was collapsed to action taken or no action*

**Fig. 2:**
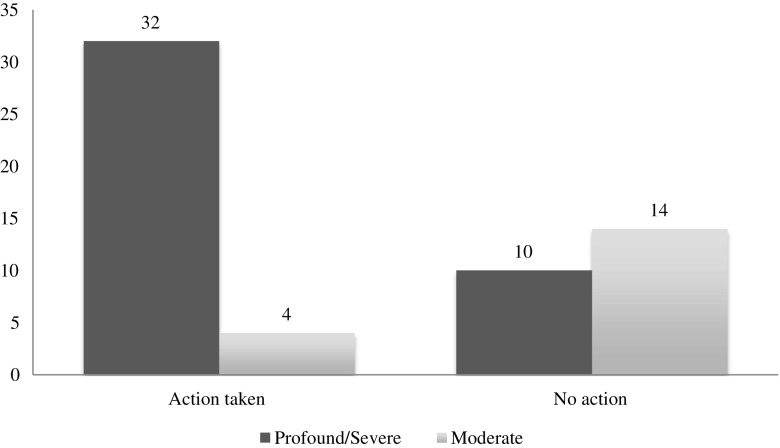
Disease severity and action taken/planned based on ECS results

Of ARC carrying severe or profound conditions, 76% (32/42) reported alternative reproductive actions, versus 22% (4/18) ARC carrying moderate conditions (Fig. 2), suggesting that disease severity has a significant effect on reproductive actions (*p* = 0.000145). One severe condition was a clear outlier to this trend: only 29% (2/7) of ARC carrying biotinidase deficiency (BTD) reported a change in their actions, in contrast to the 86% (30/35) of ARC carrying other severe or profound conditions (*p* = 0.0049). Of ARC carrying profound conditions, 80% (8/10) reported alternative reproductive actions vs. 75% (24/32) of ARC carrying severe conditions (*p* = 1.00).

Because ECS includes all of the conditions recommended by ACOG and ACMG, the data allow us to compare ARC actions between those diseases with guidelines and those without. For diseases recommended for panethnic screening (CF and spinal muscular atrophy (SMA)), 14 ARC reported taking action on the results while two did not alter their reproductive plans. There was no significant difference (*p* = 0.270) between rate of action on these recommended conditions as compared to the other severe or profound conditions, with 18 in that group reporting action based on the result and eight reporting no change to reproductive plans (Table 4). If all diseases with guidelines, including ethnicity based screening, are compared, 17 reported taking action based on the result, while three did not. Here again, there was no significant difference (*p* = 0.284) between ARC carrying diseases with screening recommendations and the ARC carrying non-guideline diseases, with 15 reporting action taken based on their results and seven reporting no change in reproductive plans.

**Table 4:** Diseases and corresponding reproductive decisions in the preconception and prenatal contexts (*N* = 64)

Disease	Severity	Fraction of preconception planning or taking action	Fraction of prenatal taking action
Smith-Lemli-Opitz Syndrome (*n* = 3)	Profound	2/2 (1 IVF + PGD)	1/1 (1 PNDx)
Carnitine Palmitoyltransferase II Deficiency (*n* = 3)	Profound	1/2 (1 IVF + PGD, 1 Unclear)	1/1 (1 PNDx)
Gaucher Disease *(n* = 1)	Profound		0/1 (1 Miscarriage)
Hereditary Fructose Intolerance (*n* = 1)	Profound		0/1
Krabbe Disease (*n* = 1)	Profound	1/1 (1 IVF + PGD)	
Medium Chain Acyl-CoA Dehydrogenase Deficiency (MCAD) (*n* = 1)	Profound	1/1 (1 IVF + PGD)	
Phenylalanine Hydroxylase Deficiency, including PKU (*n* = 1)	Profound	1/1 1 (IVF + PGD)	
Cystic Fibrosis (*n* = 15)	Severe	9/9 (7 IVF + PGD, 2 PNDx)	4/6 (4 PNDx, 1 Miscarriage)
Biotinidase Deficiency (*n* = 9)	Severe	1/6 (1 IVF + PGD, 2 Unclear)	1/3 (1 PNDx)
Familial Mediterranean Fever (*n* = 4)	Severe	2/2 (1 IVF + PGD, 1 PNDx)	1/2 (1 PNDx)
Hb Beta Chain-Related Hemoglobinopathy, including Beta Thalassemia and Sickle Cell Disease (*n* = 3)	Severe	3/3 (2 IVF + PGD, 1 PNDx)	
Short Chain Acyl-CoA Dehydrogenase Deficiency (*n* = 1)	Severe	1/1 (PNDx)	
Spinal Muscular Atrophy (*n* = 1)	Severe	1/1 (1 IVF + PGD)	
Wilson Disease (*n* = 1)	Severe	1/1 (1 IVF + PGD)	
Achromatopsia (*n* = 1)	Moderate	0/1	
Alpha-1 Antitrypsin Deficiency (*n* = 8)	Moderate	1/5 (1 IVF + PGD, 1 Unclear)	0/3
GJB2-related DFNB1 Nonsyndromic Hearing Loss and Deafness (*n* = 9)	Moderate	2/8 (2 IVF + PGD)	0/1
Glycogen Storage Disease Type V (*n* = 1)	Moderate	1/1 (1 PNDx)	

While additional options were available to the non-pregnant couples, pregnancy status was not found to be a significant variable (*p* = 0.088). Existence of prior children (*p* = 0.181), occurrence of prior miscarriages (*p* = 1.00), attainment of graduate education by at least one member of the couple (*p* = 0.590), and annual income greater than or equal to US$100,000 (*p* = 0.093) were factors not found to have significant associations.

### Actions in the Prenatal Context

Nineteen of 64 (30%) ARC were pregnant when they received ECS results. Of these, 42% (8/19) elected prenatal diagnosis (CVS or amniocentesis) and 58% (11/19) did not. Of the latter group, two participants reported planning a diagnostic procedure but miscarried before the procedure could be done, effectively bringing those who took action or planned to take action to 53% (10/19) and those who did not plan to take action to 47% (9/19). Both of the ARC who experienced a miscarriage prior to planned prenatal diagnosis indicated that they would pursue IVF with PGD in future pregnancies. Of the eight ARC who underwent prenatal diagnosis, five fetuses did not inherit both parental mutations, and three were homozygous or compound heterozygous for the parental mutations, consistent with Hardy-Weinberg expectations (*p* = 0.422, exact binomial test). Two of the latter three pregnancies were voluntarily terminated (carnitine palmitoyltransferase II deficiency, OMIM *600650, and cystic fibrosis, OMIM #219700) and one was continued (cystic fibrosis).

### Actions in the Preconception Context

Forty-five of 64 (70%) ARC were not pregnant when they received their results. Of these, 62% (28/45) responded that they pursued or planned to pursue alternative reproductive options, either IVF with PGD (*n* = 22) or prenatal diagnosis (*n* = 6). None selected the other options: gamete donation, adoption, no longer planning to have children. Twenty-nine percent (13/45) responded that they were not planning to pursue any alternative options based on the results. The remaining 9% (4/45) selected “other” and/or provided responses that were not indicative of a clear future direction. Of these 45 ARC, 31 had carrier screening as part of a fertility work-up. All ARC who did not pursue or plan to pursue alternative options carried moderate severity conditions (achromatopsia OMIM #262300, alpha-1 antitrypsin deficiency OMIM #613490, and *GJB2*-related DFNB1 nonsyndromic hearing loss and deafness OMIM #220290) or biotinidase deficiency (OMIM #253260), classified as a severe condition (see Discussion about genotype-phenotype spectrum in this condition).

### Qualitative Analysis of Decision Making

Respondents had the opportunity to provide free-text responses. Those who were not pregnant at the time of screening were asked about their reproductive choices/plans and presented with the opportunity to respond to the prompt “What factors influenced your decision?” ARC who were pregnant at the time of screening and who did not elect to pursue prenatal diagnosis were asked “What were some reasons you chose not to pursue prenatal diagnostic testing?” Those same respondents were asked about their reproductive choices/plans in future pregnancies now that they had received ECS results in response to the prompt “What factors influenced your decision?” Thematic analysis was performed on these short, free-text responses. Some responses contained more than one theme.

Free-text responses from 17 ARC discussed reasons why they choose not to pursue alternative reproductive options, with the dominant theme (14/17) being disease severity. These ARC indicated that they did not perceive the condition to be serious enough to warrant a change in reproductive planning:

*The carrier screening results we received were not linked with (in our perception) substantial pain or suffering a child might experience, and therefore not worth trying to prevent through an alternative conception/adoption option.* (Preconception; *GJB2*-related DFNB1 Nonsyndromic Hearing Loss and Deafness)

Others perceived a low risk of an affected child (3/17) with some referencing the risk from a low penetrance or mild allele (2/17). One referenced the risks of miscarriage inherent to prenatal diagnosis and another the cost of alternatives like IVF.

Of 21 ARC who provided free-text responses with their reasons for choosing to pursue alternative reproductive options, the dominant theme (11/21) also regarded severity. In this analysis, desire for a child without the disease was coded as a subtheme of disease severity.

*Symptoms and severity of the condition, if inherited and the desire to not have an affected child.* (Preconception; Hb Beta Chain-Related Hemoglobinopathy).

Several referenced the risk or chance of an affected child, indicating that they perceived the risk to be high and/or were unwilling to take the risk (6/21). Some respondents explored the choice to pursue IVF with PGD as a way to avoid the potential for the emotional pain of terminating a wanted pregnancy after prenatal diagnosis (3/21). Some indicated that they were already considering IVF and chose to add PGD after receiving the results to improve chances for a healthy pregnancy (4/21).

## Discussion

The data demonstrate that carrier screening results affected clinical decision making for the majority of these ARC. Couples who were carriers of a disease classified as profound or severe were significantly more likely to take action based on the results than those who were carriers of a moderate condition. The qualitative analysis of participants’ open-text responses describing their decision-making rationale corroborated the quantitative analysis: the majority of responses concerned perceived disease severity. Inclusion of a condition in professional society guidelines did not affect the likelihood of changed reproductive decisions.

This study also raises questions about the spectrum of variant-specific effects within recessive diseases and how this may impact reproductive decision making. Only two of nine ARC in our data carrying biotinidase deficiency reported changing their reproductive actions (two others provided unclear responses). The sharp contrast to other severe conditions raised questions as to the source of the discrepancy. While complete BTD is classified as a severe condition, partial deficiency (10–30% enzyme activity) is associated with a mild presentation. One variant, p.Asp444His (D444H, HGVS NC_000003.11:g.15686693G > C) occurs at high frequency (allele frequency of 3.94% = carrier frequency of 7.6% among non-Finnish Europeans in ExAC) (ExAC 2016; Lek et al. 2016). It is a common cause of partial BTD when combined with a classic mutation, and is associated with approximately 50% enzyme activity in homozygotes (Swango et al. 1998), similar to the asymptomatic carrier state. While participants were asked to report the condition they carried, they were not asked to report the specific variants, making it impossible to directly ascertain which ARC carried D444H. However, a review of the records of the original ARC invited to the study (including both respondents and nonrespondents) revealed that in 93% of BTD ARC (53/57), both members of the couple were D444H carriers. Our finding that most (7/9) BTD ARC did not report action taken based on this result is consistent with the hypothesis that all such ARC were D444H double-carriers (*p* = 0.19). This finding raises the issue of variant-specific effects within genetic conditions that has been well characterized in diseases such as cystic fibrosis. These results suggest a need for additional granularity in disease classification based on known genotype/phenotype correlations within a given condition.

Questions have been raised regarding provider and patient ability to sufficiently understand a wide variety of rare diseases to make informed decisions based on their carrier status (Henneman et al. 2016). One common caution regarding ECS is the overwhelming amount of information needed to counsel on each condition on the panel (Umbarger et al. 2013). Based on the clear differential in reproductive decisions between profound/severe and moderate conditions presented here, it appears that patients understood the relative level of severity and impact of the various diseases identified by ECS. This may also be a result of the high uptake of post-test genetic counseling: 95% of ARC (61/64) pursued genetic counseling after receiving their results.

### Study Limitations and Research Recommendations

In any survey research study where only a subset of eligible participants respond to the invitation, the data are not based on random sampling and may be affected by response bias. Accuracy may be limited by participants’ memory and levels of medical literacy. Couples in the preconception setting were asked what options they pursued or planned to pursue; planned behaviors may not correlate to future actions. Because of the limitations of survey response data and the limited offering of ECS outside of the fertility treatment context, caution may be needed in applying the results of this study to the general population. Our sample was disproportionately highly educated with high incomes and many respondents derived from infertility settings, characteristics that may lower the barriers to considering PGD. Future research should differentiate between the mutations carried by couples and the probable genotype/phenotype correlations that affect disease severity.

## Conclusion

This study reports the reproductive decisions made by ARC after receiving ECS results to evaluate and demonstrate the clinical utility of ECS. Not only did the majority of ARC identified through ECS alter their reproductive decisions based on these results, but there was no significant difference between the rate of action between severe and profound diseases currently recommended by professional societies and those not yet included in screening guidelines.

## Electronic supplementary material

ESM 1 (DOCX 146 kb)
