# Supplementary material for: Clinical Utility of Expanded Carrier Screening: Reproductive Behaviors of At-Risk Couples
Source: J Genet Couns. 2017 Sep 27;27(3):616–25. doi: 10.1007/s10897-017-0160-1 (PMC5943379; doi:10.1007/s10897-017-0160-1)
Supplement: Supplementary file 1 — (DOCX 146 kb) [file 10897_2017_160_MOESM1_ESM.docx]

**SUPPLEMENTARY MATERIAL**

**S1. Informed Consent**

Thank you for considering participation in this survey. This research project is being conducted by Caroline Ghiossi in partial fulfillment of a Master’s degree in Genetic Counseling at California State University Stanislaus and in collaboration with Counsyl as part of a student internship.

We hope to learn about patient experiences and decisions after receiving carrier screening results. Specifically, what options do patients pursue after learning that both they and their partner are carriers for a condition and what factors are associated with decision-making. If you decide to participate, you will be asked to complete an online survey containing approximately 25 questions about your demographic background, fertility background, and preferences regarding reproductive options. The survey is expected to take about 10-15 minutes to complete.

Your participation is voluntary and you may discontinue at any point. Please note that you can decline to answer any questions you do not wish to answer. The answers from your survey will not be connected to your name or any other identifying information. Survey responses are anonymous and all data collected will be de-identified.

There are no risks to you for your participation in this study, except for the potential emotional distress that may be caused by answering questions about your pregnancy history, your feelings regarding reproductive options, and your future family planning. Should you experience any adverse reactions to taking the survey, you may notify the principal investigator, who will put you into contact with a licensed genetic counselor. It is possible that you will not benefit directly by participating in this study; however the study may improve the genetic counseling of future patients and inform decisions regarding the inclusion of other conditions in carrier screening programs.

This study has been approved by the CSU Stanislaus University Institutional Review Board (UIRB). Any questions or concerns may be addressed to me, Caroline Ghiossi ([cghiossi@csustan.edu](mailto:cghiossi@csustan.edu)), my faculty sponsor, Dr. Janey Youngblom ([jyoungblom1@csustan.edu](mailto:jyoungblom1@csustan.edu)), or the co-investigator Kenny Wong, MS CGC (kenny@counsyl.com). If you have any questions regarding your rights and participation as a research subject, please contact the Campus Compliance Officer by phone (209) 667-3794 or email [IRBAdmin@csustan.edu](mailto:IRBAdmin@csustan.edu).

- Yes, I consent to participate [Begin Survey]
- No, I decline to participate [Exit Survey]

**S2. Survey Instrument**

*Each participant saw a subset of the questions based on their individual responses. Display, skip, and branch logic is indicated with parentheses where applicable.*

1. What factors influenced your decision to pursue carrier screening?

1. The test was part of routine screening
2. The test was part of a fertility work-up
3. My partner and I are related
4. Ethnic background
5. Family history of a genetic condition on the carrier screening test
6. Other: *[Text Box]*

2. How was your carrier screening process initiated?

1. A healthcare provider recommended carrier screening
2. I asked a healthcare provider to order carrier screening
3. Other: *[Text Box]*

3. How long ago did you receive your carrier screening results?

1. 1-3 months
2. 3-6 months
3. 6-9 months
4. More than 9 months

4. Select the condition that both you and your partner were found to carry: *[Drop-down Menu]*

1. 98 conditions included in the study*
2. My partner and I were not carriers for the same condition
3. I don’t recall

5. Did you pursue genetic counseling to discuss your results?

1. Yes, with genetic counselor at Counsyl
2. Yes, with a local genetic counselor
3. Yes, with a healthcare provider other than a genetic counselor
4. No, but I may consider it in the future
5. No, I don’t plan to pursue genetic counseling

5:AB. *[If “Yes” to genetic counselor at Counsyl or local genetic counselor is selected]* Please read each statement below and select the option that best represents your experience with genetic counseling *[5-point Likert scale from strongly disagree to strongly agree]^[[1]](#footnote-1)^*:

- My genetic counselor seemed to understand the stresses I was facing
- My genetic counselor helped me to identify what I needed to know to make decisions
- The genetic counseling session was the right length of time I needed
- My genetic counselor was truly concerned about my well-being
- The genetic counseling session was valuable to me

5:DE. *[If either “No” response is selected]* What are some reasons you decided not to pursue genetic counseling? *[Text Box]*

6. Were you pregnant when you received your carrier screening results?

1. Yes
2. No

*Survey Branch Point:*

*-If “Yes” the questions in the “Prenatal” block of questions will be displayed.*

*-If “No” the questions in the “Preconception” block of questions will be displayed.*

*-If the respondent declines to answer the question, the “Decline” block of questions will be displayed.*

[Decline Block: *If the participant declines to answer question number 6 and neither option is selected*]

7. After receiving your carrier screening results, what option(s) did you or are you planning to pursue?

1. In vitro fertilization (IVF) with preimplantation genetic diagnosis (PGD) for the condition in question
2. Sperm or egg donation
3. Adoption
4. Prenatal diagnostic testing such as chorionic villus sampling (CVS) or amniocentesis
5. No longer planning to have children
6. Not planning to pursue any alternative options
7. Other: *[Text Box]*

8. What factors influenced your decision? *[Text Box]*

[Preconception Block: *If “No” is selected for question number 6, indicating that the participant was not pregnant at the time of receiving results*]

9. Which of the following best describes your situation at the time you received your carrier screening results?

1. Actively trying to conceive, not using any form of contraception
2. Thinking about trying to conceive in the next 6 months
3. Thinking about trying to conceive in the next year
4. No specific plans to try to conceive
5. Other: *[Text Box]*

10. After receiving your carrier screening results, what option(s) did you or are you planning to pursue?

1. In vitro fertilization (IVF) with preimplantation genetic diagnosis (PGD) for the condition in question
2. Sperm or egg donation
3. Adoption
4. Prenatal diagnostic testing such as chorionic villus sampling (CVS) or amniocentesis
5. No longer planning to have children
6. Not planning to pursue any alternative options
7. Other: *[Text Box]*

11. What factors influenced your decision? *[Text Box]*

[Prenatal Block: *If “Yes” is selected for question number 6, indicating that the participant was pregnant at the time of receiving results*]

12. How many weeks was your pregnancy at the time you received your carrier screening results?

1. 0-13 weeks
2. 14-26 weeks
3. 27-40 weeks

13. After receiving your carrier screening results, did you pursue prenatal diagnostic testing such as chorionic villus sampling (CVS) or amniocentesis for the condition?

1. No

13.A. *[If “No” is selected]* What were some reasons you chose not to pursue prenatal diagnostic testing? *[Text Box]*

1. Yes

13.B. *[If “Yes” is selected]* What did the final test result show?

- 1. The pregnancy was not found to be affected with the condition
  2. The pregnancy was found to be affected with the condition

13.B.b. *[If “The pregnancy was found to be affected with the condition” is selected]* The outcome after learning the final test results was that:

- - 1. The pregnancy was terminated
    2. The pregnancy was continued
    3. The pregnancy miscarried

13.B.b.i *[If any response to 13.B.b.i. is selected]* Is there any part of your experience you would like to share? *[Text Box]*

14. What option(s) do you plan to pursue in the future?

1. In vitro fertilization (IVF) with preimplantation genetic diagnosis (PGD) for the condition in question
2. Sperm or egg donation
3. Adoption
4. Prenatal diagnostic testing such as chorionic villus sampling (CVS) or amniocentesis
5. No longer planning to have children
6. Not planning to pursue any alternative options
7. Other: *[Text Box]*

15. What factors influenced your decision? *[Text Box]*

*[Survey Conjoin Point: All participants will be shown the following block of demographic questions]*

16. What was the age of the female partner at the time of screening?

1. 18-24
2. 25-34
3. 35-44
4. 45 or over

17. How many children do you have at this time?

1. None
2. 1
3. 2
4. 3 or more

18. How many times have you been pregnant? *[Text Box]*

19. Have you had any miscarriages?

1. Yes
2. No

20. What is your ethnicity? (select all that apply)

1. Northern European e.g. British, German
2. Southern European e.g. Italian, Greek
3. French Canadian or Cajun
4. Ashkenazi Jewish
5. Other/Mixed Caucasian
6. East Asian e.g. Chinese, Japanese
7. South Asian e.g. Indian, Pakistani
8. Southeast Asian e.g. Filipino, Vietnamese
9. African or African American
10. Hispanic
11. Middle Eastern
12. Native American
13. Pacific Islander
14. Unknown
15. Other: *[Text Box*

21. What is your partner’s ethnicity? (select all that apply)

1. Northern European e.g. British, German
2. Southern European e.g. Italian, Greek
3. French Canadian or Cajun
4. Ashkenazi Jewish
5. Other/Mixed Caucasian
6. East Asian e.g. Chinese, Japanese
7. South Asian e.g. Indian, Pakistani
8. Southeast Asian e.g. Filipino, Vietnamese
9. African or African American
10. Hispanic
11. Middle Eastern
12. Native American
13. Pacific Islander
14. Unknown
15. Other: *[Text Box]*

22. What is your religious affiliation?

1. Rather not say
2. Protestant
3. Catholic
4. Mormon
5. Jewish
6. Buddhist
7. Hindu
8. Muslim
9. No religious affiliation
10. Other: *[Text Box]*

23. What is your partner’s religious affiliation?

1. Rather not say
2. Protestant
3. Catholic
4. Mormon
5. Jewish
6. Buddhist
7. Hindu
8. Muslim
9. No religious affiliation
10. Unknown
11. Other: *[Text Box]*

24. What is the highest level of education you completed?

1. Elementary School
2. High School or equivalent
3. Vocational/Technical School
4. Some College
5. Associate Degree
6. Bachelor Degree
7. Graduate Degree

25. What is the highest level of education your partner completed?

1. Elementary School
2. High School or equivalent
3. Vocational/Technical School
4. Some College
5. Associate Degree
6. Bachelor Degree
7. Graduate Degree
8. Unknown

26. What is your current household income in U.S. dollars?

1. Rather not say
2. Under $20,000
3. $20,000 - $29,999
4. $30,000 - $39,999
5. $40,000 - $49,999
6. $50,000 - $74,999
7. $75,000 - $99,999
8. $100,000 - $150,000
9. Over $150,000

*[End of survey text]* We thank you for your time spent taking this survey. Your response has been recorded.

**Full list of conditions available in the drop-down menu for question number 4:*

- ABCC8-related Hyperinsulinism
- Achromatopsia
- Alkaptonuria
- Alpha-1 Antitrypsin Deficiency
- Alpha-Mannosidosis
- Andermann Syndrome
- ARSACS
- Aspartylglycosaminuria
- Ataxia With Vitamin E Deficiency
- Ataxia-Telangiectasia
- Autosomal Recessive Polycystic Kidney Disease
- Bardet-Biedl Syndrome, BBS1-related
- Bardet-Biedl Syndrome, BBS10-related
- Biotinidase Deficiency
- Bloom Syndrome
- Canavan Disease
- Carnitine Palmitoyltransferase IA Deficiency
- Carnitine Palmitoyltransferase II Deficiency
- Cartilage-Hair Hypoplasia
- Citrullinemia Type 1
- CLN3-related Neuronal Ceroid Lipofuscinosis
- CLN5-related Neuronal Ceroid Lipofuscinosis
- Cohen Syndrome
- Congenital Disorder of Glycosylation Type Ia
- Congenital Disorder of Glycosylation Type Ib
- Congenital Finnish Nephrosis
- Costeff Optic Atrophy Syndrome
- Cystic Fibrosis
- Cystinosis
- D-Bifunctional Protein Deficiency
- Factor XI Deficiency
- Familial Dysautonomia
- Familial Mediterranean Fever
- Fanconi Anemia Type C
- Galactosemia
- Gaucher Disease
- GJB2-related DFNB1 Nonsyndromic Hearing Loss and Deafness
- Glutaric Acidemia Type 1
- Glycogen Storage Disease Type Ia
- Glycogen Storage Disease Type Ib
- Glycogen Storage Disease Type III
- Glycogen Storage Disease Type V
- GRACILE Syndrome
- Hb Beta Chain-Related Hemoglobinopathy (Including Beta Thalassemia and Sickle Cell Disease)
- Hereditary Fructose Intolerance
- Hereditary Thymine-Uraciluria
- Herlitz Junctional Epidermolysis Bullosa, LAMA3-related
- Herlitz Junctional Epidermolysis Bullosa, LAMB3-related
- Herlitz Junctional Epidermolysis Bullosa, LAMC2-related
- Hexosaminidase A Deficiency (Including Tay-Sachs Disease)
- Homocystinuria Caused by Cystathionine Beta-Synthase Deficiency
- Hurler Syndrome
- Hypophosphatasia, Autosomal Recessive
- Inclusion Body Myopathy 2
- Isovaleric Acidemia
- Joubert Syndrome 2
- Krabbe Disease
- Limb-Girdle Muscular Dystrophy Type 2D
- Limb-Girdle Muscular Dystrophy Type 2E
- Lipoamide Dehydrogenase Deficiency
- Long Chain 3-Hydroxyacyl-CoA Dehydrogenase Deficiency
- Maple Syrup Urine Disease Type 1B
- Medium Chain Acyl-CoA Dehydrogenase Deficiency
- Megalencephalic Leukoencephalopathy With Subcortical Cysts
- Metachromatic Leukodystrophy
- Mucolipidosis IV
- Muscle-Eye-Brain Disease
- NEB-related Nemaline Myopathy
- Niemann-Pick Disease Type C
- Niemann-Pick Disease, SMPD1-associated
- Nijmegen Breakage Syndrome
- Northern Epilepsy
- Pendred Syndrome
- PEX1-related Zellweger Syndrome Spectrum
- Phenylalanine Hydroxylase Deficiency (Including PKU)
- Polyglandular Autoimmune Syndrome Type 1
- Pompe Disease
- PPT1-related Neuronal Ceroid Lipofuscinosis
- Primary Carnitine Deficiency
- Primary Hyperoxaluria Type 1
- Primary Hyperoxaluria Type 2
- PROP1-related Combined Pituitary Hormone Deficiency
- Pycnodysostosis
- Rhizomelic Chondrodysplasia Punctata Type 1
- Salla Disease
- Segawa Syndrome
- Short Chain Acyl-CoA Dehydrogenase Deficiency
- Sjogren-Larsson Syndrome
- Smith-Lemli-Opitz Syndrome
- Spinal Muscular Atrophy
- Steroid-Resistant Nephrotic Syndrome
- Sulfate Transporter-Related Osteochondrodysplasia
- TPP1-related Neuronal Ceroid Lipofuscinosis
- Tyrosinemia Type I
- Usher Syndrome Type 1F
- Usher Syndrome Type 3
- Very Long Chain Acyl-CoA Dehydrogenase Deficiency
- Wilson Disease

1. Adapted from Demarco TA, Peshkin BN, Mars BD, Tercyak KP. Patient Satisfaction with Cancer Genetic Counseling: A Psychometric Analysis of the Genetic Counseling Satisfaction Scale. *J Genet Couns.*2004;13(4):293-304. doi:10.1023/b:jogc.0000035523.96133.bc. [↑](#footnote-ref-1)
